# Factors Associated with HIV Status Disclosure to Orphans and Vulnerable Children Living with HIV: Results from a Longitudinal Study in Tanzania

**DOI:** 10.1155/2020/6663596

**Published:** 2020-12-27

**Authors:** Shraddha Bajaria, Amon Exavery, Noreen Toroka, Asheri Barankena, John Charles, Levina Kikoyo

**Affiliations:** ^1^Ifakara Health Institue, P. O. Box 78373, Dar es Salaam, Tanzania; ^2^Pact, P.O.Box 6348, Dar es Salaam, Tanzania

## Abstract

**Background:**

The Tanzanian national guideline for pediatric HIV disclosure recommends beginning disclosure as early as age 4–6 years; full disclosure is recommended at the age of 8–10 years. Despite clear procedures, the disclosure rate in Tanzania remains relatively low. This study assessed the factors associated with HIV status disclosure to orphans and vulnerable children living with HIV (OVCLHIV).

**Methods:**

Data for this analysis come from the USAID-funded Kizazi Kipya program in Tanzania that provides health and social services to OVC and caregivers of HIV-affected households. Data were collected between January 2018 and March 2019. Disclosure status was self-reported by caregivers of children aged 8 years or above. Beneficiary characteristics were included as independent variables. Generalized estimating equations took into account the clustering effect of the study design.

**Results:**

Of the 10673 OVCLHIV, most were females (52.43%), and 80.67% were enrolled in school. More than half (54.89%) were from households in rural areas. Caregivers were mostly females (70.66%), three quarters were between 31 and 60 years old and had a complete primary education (67.15%), and 57.75% were HIV-infected. Most of the OVCLHIV (87.31%) had a disclosed HIV status. Greater OVCLHIV age (*p* < 0.001), school enrollment (OR = 1.22; 95% CI 1.06, 1.41), urban location of household (OR = 1.64; 95% CI 1.44, 1.86), caregivers' higher education level (*p* < 0.001), and caregiver HIV-positive status (OR = 1.25; 95% CI 1.09, 1.43) were positively associated with disclosure status. OVCLHIV of female caregivers were 27% less likely to have been disclosed than those of male caregivers.

**Conclusion:**

The disclosure rate among OVCLHIV in this study was high. Disclosure of HIV status is crucial and beneficial for OVCLHIV continuum of care. Caregivers should be supported for the disclosure process through community-based programs and involvement of health volunteers. Policymakers should take into consideration the characteristics of children, their caregivers, and location of households in making disclosure guidelines as adaptable as possible.

## 1. Background

Globally, about 1.8 million children under the age of 15 years were living with HIV in 2019 [1], and most of whom were in eastern and southern Africa. The UNAIDS 90-90-90 target for 2020 aimed for 90% of all people living with HIV (PLHIV) to know their status, 90% of all people diagnosed with HIV to receive sustained antiretroviral therapy (ART), and 90% of all people receiving ART to have viral suppression. In 2018, 78% of all PLHIV in Tanzania were aware of their status, of which 92% were receiving ART and 87% were virally suppressed. Of all adults aged 15 years or above, 72% were on ART while only 65% of all children under the age of 15 years living with HIV were on ART [2]. Achieving the continuum of care can be difficult among HIV-infected children who are dependent on their parents, caregivers, or health workers to look after them. The literature shows that lower proportions of children in low- and middle-income countries know their status compared to their counterparts in higher-income countries [3]. In many countries and cultures, HIV continues to remain a stigmatized and sensitive topic [4], and parents or caregivers are often uncertain on how to counsel about disclosure. However, delaying the disclosure process could adversely affect child outcomes, including access and adherence to HIV care and viral suppression [5].

The Tanzanian national guidelines for the management of HIV recommend pediatric HIV disclosure to be done over time, beginning at the age of 4–6 years [6]. The child can be informed to have a chronic illness that requires regular clinic visits and medications. Full status disclosure is recommended at the age of 8–10 years, in a caring and supportive manner. It is recommended that adolescents and school-going children should know their HIV status, how HIV is spread, and how to stay healthy, in order to make them responsible for their own health and well-being [5, 6]. Despite these guidelines, the disclosure rate among children in Tanzania remains low. In 2017, only 50.2% of HIV-infected young people between ages of 15 and 24 years were aware of their status, compared to 60.6% of all PLHIV aged above 15 years [7]. HIV prevalence among children aged 0–14 years was 0.4%, and the overall viral load suppression, irrespective of awareness of their own HIV-positive status, was 18.4% [7].

Some factors that reportedly influence HIV disclosure to children include child's age [3, 8, 9], being an orphan [8], and caregiver characteristics such as educational level and older age, caregivers' knowledge about HIV, and caregivers' monthly income [10, 11]. Many studies have identified challenges faced by caregivers in disclosing children's HIV status to them such as fear of possible adverse outcomes, uncertainty of child's reaction, psychological harm, stigma, or trauma [3, 8, 12]. However, some qualitative studies [13, 14] have also captured caregivers' perception on positive outcomes of disclosing child's HIV status, such as improved adherence to treatment, improved caregiver-child relationship, better understanding of potential risks by the child, and access to social support.

While a handful of studies have so far looked at the factors associated with disclosure of HIV status to their children in general population settings, fewer have considered orphans and vulnerable children (OVC) groups. Understanding child and caregiver characteristics as well as other factors that influence HIV status disclosure to children is necessary in planning for guidelines and interventions to support vulnerable children [8]. This study aims to assess factors associated with HIV disclosure of OVC living with HIV (OVCLHIV) in Tanzania. Specifically, the study explores the association between the HIV disclosure status of OVCLHIV and caregiver and child characteristics. The study also examines differences in disclosure status between urban and rural areas and differences in disclosure status within a household of a single caregiver looking after multiple OVCLHIV, for beneficiaries who are enrolled in the United States Agency for International Development- (USAID-) funded Kizazi Kipya program in Tanzania. This community-based five-year program (2016–2021) aims to provide opportunities for utilization of HIV services, reduce barriers to access and uptake of HIV services, and ensure tracking of beneficiaries at the household level and directly providing services such as treatment adherence, HIV prevention education, and status disclosure support and facilitating bidirectional referral for other HIV services through the national referral system for OVCLHIV and their caregivers. The program also aims to improve other health, nutrition, education, protection, economic, and social outcomes for OVC and their caregivers, especially those affected by HIV.

## 2. Methods

### 2.1. Study Design and Data Source

This cross-sectional secondary analysis used existing monitoring data from the Kizazi Kipya program in Tanzania. Potential beneficiaries are identified by the district social welfare officials, child protection teams, schools, faith-based groups or from care and treatment clinics (CTC), health facilities, or by community health workers (CHWs). During the screening and enrollment of beneficiaries, OVC caregivers self-report reasons for household vulnerability; households meeting the project criteria are then enrolled. The project criteria are detailed elsewhere [15]. After the eligible beneficiaries' consent to be enrolled in the program, the needs of the household are identified and case-specific care plans are developed by community workers. Following this, the family and child asset assessment (FCAA) is conducted, to evaluate the household's needs, the primary caregivers' characteristics, and health, social, and household economic statuses, in order to develop a specific care plan. The program's data collection tool, the HIV risk assessment and adherence form, administered by community caseworkers, collects data on OVC risk of infection, latest HIV status and treatment adherence, enrollment into CTC, and whether a referral is needed. This form is completed once a year, and all responses are self-reported by the caregiver of OVC.

### 2.2. Study Area and Population

This study used data from 18 regions of Tanzania, collected between January 2018 and March 2019. Caregivers of OVC aged between 0 and 19 years respond to the HIV risk assessment and adherence form. HIV status disclosure to OVCLHIV aged 8 years or above is enquired from their caregivers through this tool. Therefore, the inclusion criteria for this study were all OVCLHIV aged 8 years or above and their caregivers, who responded to the HIV risk assessment and adherence form during the specified time period.

### 2.3. Details of Measurement

HIV status disclosure, collected during the HIV risk and adherence assessment, was the outcome variable for this study. This question on the form is only answered if the child or adolescent is HIV-positive and 8 years or above and the caregiver self-reports the status.

OVC and caregiver characteristics were included as the independent variables in this study. All of these data come from the screening, enrollment, and FCAA data. OVC age groups were categorized as 8 to 10 years, 11 to 14 years, and 15 years or above. OVC school enrollment status recorded whether the child was attending school at the time of data collection. Caregiver ages were categorized as 18 to 30 years, 31 to 60 years, and above 60 years. The education level of caregivers was characterized as never attended, primary incomplete, primary complete, and postprimary. Caregiver marital status was classified as never been married, married or cohabiting, divorced, separated or widowed, or others. Councils were categorized as urban if they were city, municipal, or town councils and rural if they were district councils.

### 2.4. Data Analysis

Data were analyzed using Stata 15 software, using OVC as the unit of analysis. To take into account the clustering effect of OVC within a household, generalized estimating equations (GEEs) with logit link function utilizing a binomial distribution family and an exchangeable correlation structure were used to analyze the OVC and caregiver characteristics that are associated with disclosure of OVC HIV status. Factors that were significantly associated in the univariable analysis were included in the multivariable model; a *p* value of <0.05 was considered significant.

For caregivers taking care of multiple OVCLHIV within the same household, sensitivity analyses were done using the chi-square test, (1) to check if there is a difference in disclosure status by any of the OVC characteristics and (2) to check if there is a difference in disclosure status by any of the caregiver characteristics. Because significant differences in disclosure by caregiver characteristics were observed, a subset of the study population, all caregivers looking after more than one OVC, was created and an outcome variable of their OVC's disclosure status was generated. The main interest was whether all their OVC have been disclosed or only some had their HIV status disclosed. GEEs were used to analyze the characteristics of OVC and caregivers that are associated with all OVC within the household knowing their HIV status. Due to the interest of the authors and factors associated with disclosure status in previously done studies, the OVC age was included as a priori in the multivariable model.

## 3. Results

### 3.1. Characteristics of Study Population

Characteristics of the OVCLHIV and caregivers included in this study are presented in Table 1. A total of 10673 HIV-positive OVC aged 8 years or above were eligible for this analysis. A quarter (24.84%) of the OVC were aged between 8 and 10 years, most were between 11 and 14 years (38.43%), and 36.73% were 15 years or above. Slightly more than half (52.43%) of the OVC were females. Most OVC (80.67%) were enrolled in school at the time of data collection, most of which were under the age of 18. Almost two-thirds (58.75%) of all OVC who were out of school were aged 15 years or above. About 55% of the OVC were from rural areas. A total of 9300 caregivers looked after the OVC included in this study, suggesting that there were some caregivers looking after multiple OVCLHIV in their households. Most caregivers were between the age of 31 and 60 years (73.61%). Most caregivers were females (70.66%), and more than two-thirds (67.15) had completed primary education. About half of the caregivers were married or cohabiting (51.44%), 40.13% were divorced or separated, and 7.13% had never been married. More than half of the caregivers were HIV-infected (57.75%).

### 3.2. Proportions of OVCLHIV Who Had Disclosed HIV Status by Location and Characteristics

Overall, disclosure among OVCLHIV aged 8 years or above was high; 87.31% of all OVCLHIV included in this analysis had a disclosed HIV status. Disclosure was significantly higher among OVCLHIV in the urban area; 90.34% of OVCLHIV in the urban versus 84.82% of OVCLHIV in the rural area had been disclosed (*p* < 0.001).  Table 2 presents the characteristics of the disclosed OVC and their caregivers by their location: urban and rural. There was no significant difference in disclosure by OVCLHIV characteristics in the urban area. In the rural area, older children were more likely to have a disclosed status than younger ones (*p* < 0.001), and there was a trend for disclosure in OVCLHIV who were enrolled in school, with a borderline *p* value of 0.049. There were significant differences in disclosure by caregiver characteristics. In the urban area, more OVCLHIV of caregivers who had completed primary education, had never been married, or were HIV-infected themselves had a disclosed HIV status. In the rural area, more OVCLHIV of caregivers who were males, with completed primary education, and positive HIV status had a disclosed HIV status.

### 3.3. OVCLHIV and Caregiver Characteristics That Are Associated with Disclosure of OVC's HIV Status

Table 3 presents the results of the GEE univariable and multivariable analyses. In the univariable model, OVCLHIV characteristics that were significantly associated with disclosure status were OVC age and their status of school enrollment. OVCLHIV who were between the age of 11 and 14 years (OR = 1.31; 95% CI (1.14, 1.51)) or 15 or above (OR = 1.42; 95% CI (1.23, 1.65)) were more likely to have a disclosed HIV status than younger OVCLHIV aged between 8 and 10 years. OVCLHIV who were enrolled in school at the time of data collection were 23% more likely to have a disclosed HIV status than those who were out of school. The association of both OVCLHIV age and OVCLHIV school enrollment status to disclosure remained the same in the multivariable model. OVC in the urban areas were significantly more likely to have a disclosed HIV status (OR = 1.64; 95% CI (1.44, 1.86)) than OVCLHIV in the rural areas.

Caregiver characteristics that were significantly associated with disclosure status in the univariable model were sex, education level, marital status, and HIV status. When adjusted for potential confounders, OVCLHIV of female caregivers were less likely to have a disclosed HIV status (OR = 0.73; 95% CI (0.63, 0.83)). OVC of caregivers with completed primary education were significantly more likely (OR = 1.44; 95% CI (1.24, 1.67)) to have their HIV status disclosed than those of caregivers who had never attained any education. OVCLHIV who had an HIV-infected caregiver were more likely to have a disclosed HIV status than those with an uninfected caregiver (OR = 1.25; 95% CI (1.09, 1.43)). Caregiver marital status was not associated with OVC's HIV disclosure status when adjusted for confounders.

### 3.4. Sensitivity Analysis and GEE Results for the Subset Population of Caregivers of Multiple OVCLHIV

Disclosure status for OVCLHIV of caregivers looking after more than one OVC was different for some households, for example, in the same household, one OVC would have a disclosed HIV status while the other would not. Chi-square test results revealed no significant differences in disclosure status for multiple OVCLHIV by their own characteristics; caregivers' decision to disclose the status of their multiple OVCLHIV was not influenced by OVC characteristics. However, caregiver characteristics were significantly associated with disclosure status of OVCLHIV.  Table 4 shows the proportion of caregivers who disclosed HIV status of all their OVC by their characteristics. Significantly more OVC in households caring for multiple OVCLHIV and led by females were likely to all be disclosed, while multiple OVCLHIV in households led by males were likely to have different disclosure status. There were significant differences in OVCLHIV disclosure status by caregivers' education level (*p*=0.006) as well as marital status (*p*=0.012).

The chi-square test results were further supported by the GEE analyses shown in Table 5. In the multivariable model, OVC age was not significantly associated with disclosure status, in households with multiple OVCLHIV. OVCLHIV in households led by female caregivers were almost twice as more likely to all be disclosed their HIV status (OR = 1.97; 95% CI (1.05, 2.05)) than those in households led by males. Caregivers of multiple OVCLHIV who had an incomplete primary education (OR = 2.72; 95% CI (1.32, 5.62)) or a postprimary education (OR = 6.79; 95% CI (1.58, 29.25)) were more likely to disclose HIV status of all their OVCLHIV than those who had never attained education. OVC in households led by caregivers who had ever been married were less likely to all be disclosed their HIV status than those living with caregivers who had never been married. OVCLHIV in households with multiple HIV-infected OVC in urban areas were more likely to all have their HIV status disclosed to them than those in households in rural areas (OR = 1.47; 95% CI (1.05, 2.05)).

## 4. Discussion

This study aimed to assess the factors associated with caregiver disclosure of HIV-positive status to OVCLHIV, for beneficiaries of a community-based program in Tanzania. The findings have shown that the implementation of a large-scale program such as the USAID Kizazi Kipya is an important intervention in increasing the rate of HIV status disclosure among OVC. Disclosure among OVC aged 8 years or above was relatively high; 87.31% of all OVCLHIV included in this study had been disclosed their HIV status.

OVC factors that were significantly associated with HIV status disclosure were their age and school enrollment status. There was a positive association between OVC age and their disclosure status. While the national guidelines recommend full status disclosure between ages 8 and 10 years [6], the study results indicate that OVCLHIV aged between 11 and 14 years were 31% and OVCLHIV aged between 15 and 18 years were 48% more likely to be told their HIV status than those aged between 8 and 10 years. This finding is consistent with previously done studies [3, 8, 16], some reporting caregivers' belief of children being too young to understand HIV-related processes and consequences [17, 18], coupled with fear of secondary disclosure by the child [19] to others. Interventions should focus on educating children about HIV-related matters from a young age and creating a safe environment so that caregivers can conduct the disclosure process for OVCLHIV with ease and children can develop awareness early in their life. Interventions should also enable caregivers and provide them with tools to help with disclosure to young children. OVCLHIV who were enrolled in school were more likely to have a disclosed HIV status than those who were not in school. Since the study population was 8 years or above, it cannot be argued that those who were out of school were young to be attending school; higher proportion of OVCLHIV who were not enrolled in school were aged 15 years or above. Therefore, caregivers of OVCLHIV who were in school most likely disclosed because of OVC's improved capacity to understand HIV-related issues and better ability to handle the disclosure process [19].

Caregiver characteristics that were significantly associated with HIV status disclosure to OVCLHIV were their sex, education level, and HIV status. Female caregivers were 27% less likely than males to disclose the status of OVCLHIV. This could be attributed to the greater emotional concern that women have for their children. A study from Zimbabwe reported grandmothers in particular worrying about the emotional fragility of their grandchildren still struggling with the death of a parent [20] and being apprehensive of their reaction if told about their positive HIV status. There is evidence that, in situations of infected mothers having to disclose their own status to their children, there is anxiety, worry, and hesitation [21, 22], as they fear the reaction of their children. Education level of caregivers was positively associated with disclosure of their OVC's HIV status. This is consistent with some findings from the literature, suggesting that caregivers with higher education feel more prepared to handle the disclosure process than those with lower education [19]; however, many other studies have reported results contrary to these [23, 24], citing higher disclosure levels by caregivers with less education. Caregivers who were HIV-positive themselves were more likely to disclose the child's HIV status. This finding is in contrast to previously done studies indicating caregivers' fears of negative reaction or anger by the child as well as concerns about the stigma related to HIV and fears of being blamed for infecting the child, if the caregiver is a biological parent [19, 25]. Consequently, some study results have also found that HIV-infected nonbiological caregivers were more likely to disclose the child's HIV status to them [10, 16, 19].

This study also found interesting results for a subset of the population, caregivers who looked after more than one OVCLHIV. Caregivers of multiple OVCLHIV in urban areas were more likely to reveal HIV-positive status to all their children, compared to their counterparts in rural areas. This could be attributed to lower HIV-related stigma and better understanding of HIV infection in the urban areas, as found in previously done studies [26, 27]. Limited studies have investigated the difference in rates of disclosure among children in urban and rural areas. A study that assessed the prevalence and factors associated with HIV status disclosure among children and adolescents selected from urban and rural clinics across western Kenya reported a higher prevalence of disclosure among children at rural clinics than those at a large referral hospital in the urban area [8]. In contrast, a study done in Zimbabwe revealed that adolescent girls and young women in rural areas learned their HIV-positive status for the first time at an older age (median of 19 years), compared to those in urban areas (median of 16 years) [28].

Interestingly, in contrast to the results for the whole study population that female caregivers were less likely to disclose HIV status of their OVC, female caregivers of multiple OVCLHIV were more likely to disclose HIV status to all OVC living in the same household, compared to their male counterparts. Caregivers of multiple OVCLHIV who had never been married or who had a higher level of education were also more likely to disclose HIV status to all their OVC.

Many previously done studies [17, 29, 30] have reported caregivers' lack of skills for disclosure and preference of involving CHWs and other community and health volunteers during the disclosure process. While the presented analysis does not provide enough evidence for this, the overall high disclosure rate could be attributed to the program intervention of CHWs providing counseling and referring caregivers for disclosure support. Future analysis should further analyze the factors associated with disclosure of OVCLHIV status in relation to the referrals and program services provided to the caregivers.

## 5. Additional Points

A major strength of this analysis is the large, country-representative study population of a highly vulnerable group: OVCLHIV and their caregivers. This study, however, did not analyze certain components that would have led to a better understanding of the disclosure rate: when disclosure was done, how long OVCLHIV had been in the project and whether caregivers received support for disclosure. This study did also not include controls. The data used for this study did not record whether the caregiver was a biological parent or a grandparent of the OVCLHIV. Future studies should take these into account and a qualitative study can aid in better understanding the community-level barriers to disclosure.

## 6. Conclusion

The results from this study highlight the importance of services provided by country-wide programs such as Kizazi Kipya that identify and support caregivers who struggle with disclosing their child's HIV status to them. Disclosure of HIV status is crucial and beneficial for OVC's continuum of care [5]; therefore, caregivers should be supported and be educated about the importance of the disclosure process. The barriers identified in this study can aid policymakers in targeting the most vulnerable groups of caregivers.

## Figures and Tables

**Table 1:** Characteristics of all OVCLHIV and their caregivers included in this study.

	Number of beneficiaries	Proportion (%)
OVC characteristics	*n* = 10673	
Age (in years)		
8–10	2651	24.84
11–14	4102	38.43
15 or above	3920	36.73

Sex		
Male	5077	47.57
Female	5596	52.43

School enrollment status		
In school	8610	80.67
Out of school	2063	19.33

Location		
Rural	5858	54.89
Urban	4815	45.11

Caregiver characteristics	*n* = 9300	
Age (in years)		
18–30	377	4.05
31–60	6846	73.61
Above 60	2077	22.33

Sex		
Male	2729	29.34
Female	6571	70.66

Education level		
Never attended	1689	18.16
Primary incomplete	927	9.97
Primary complete	6245	67.15
Postprimary	439	4.72

Marital status		
Never been married	663	7.13
Married/cohabiting	4784	51.44
Divorced/separated or widowed	3732	40.13
Others	121	1.30

HIV status		
Positive	5371	57.75
Negative	2504	26.92
Unknown	1425	15.32

**Table 2:** Differences in HIV status disclosure by location and OVC and caregiver characteristics.

	Urban			Rural		
	*n*	%	*p* value	*n*	%	*p* value
OVC characteristics						
Age (in years)			0.398			<0.001
8–10	983	89.28		1259	81.23	
11–14	1614	90.67		1988	85.62	
15 or above	1753	90.64		1722	86.71	

Sex			0.275			0.331
Male	2052	90.84		2377	83.35	
Female	2298	89.91		2592	85.26	

School enrollment status			0.074			0.049
In school	3579	90.70		3978	85.29	
Out of school	771	88.72		991	83.00	

Caregiver characteristics						
Age (in years)			0.869			0.470
18–30	216	89.63		147	81.67	
31–60	3248	90.30		3652	84.85	
Above 60	886	90.69		1170	85.15	

Sex			0.186			<0.001
Male	886	91.43		1870	87.02	
Female	3464	90.07		3099	83.55	

Education level			<0.001			<0.001
Never attended	529	85.05		1062	80.95	
Primary incomplete	400	88.89		507	84.78	
Primary complete	3094	91.46		3279	86.24	
Postprimary	327	90.83		121	81.88	

Marital status			<0.001			0.796
Never been married	472	92.55		212	82.81	
Married/cohabiting	1874	88.86		2846	84.90	
Divorced/separated or widowed	1945	91.79		1857	84.99	
Others	59	76.62		54	83.08	

HIV status			<0.001			<0.001
Positive	2557	92.61		2941	86.02	
Negative	1160	88.55		1319	84.93	
Unknown	633	85.08		709	80.02	

**Table 3:** GEE results of OVC and caregiver characteristics associated with disclosure status of OVCLHIV.

	Univariable (*n* = 10673)		Multivariable (*n* = 10673)	
	OR	95% CI	OR	95% CI
OVC characteristics				
Age (in years)				
8–10	1.0		1.0	
11–14	1.31^*∗∗∗*^	(1.14, 1.51)	1.33^*∗∗∗*^	(1.15, 1.53)
15 or above	1.42^*∗∗∗*^	(1.23, 1.65)	1.48^*∗∗∗*^	(1.28, 1.72)

Sex				
Male	1.0			
Female	1.01	(0.90, 1.14)		

School enrollment status				
Out of school	1.0		1.0	
In school	1.23^*∗∗*^	(1.07, 1.41)	1.22^*∗∗*^	(1.06, 1.41)

Location				
Rural	1.0		1.0	
Urban	1.68^*∗∗∗*^	(1.49, 1.89)	1.64^*∗∗∗*^	(1.44, 1.86)

Caregiver characteristics				
Age (in years)				
18–30	1.0			
31–60	1.10	(0.83, 1.46)		
Above 60	1.11	(0.82, 1.51)		

Sex				
Male	1.0		1.0	
Female	0.87^*∗*^	(0.76, 0.99)	0.73^*∗∗∗*^	(0.63, 0.83)

Education level				
Never attended	1.0		1.0	
Primary incomplete	1.39^*∗∗*^	(1.13, 1.72)	1.22	(0.99, 1.53)
Primary complete	1.69^*∗∗∗*^	(1.47, 1.94)	1.44^*∗∗∗*^	(1.24, 1.67)
Postprimary	1.61^*∗∗∗*^	(1.20, 2.16)	1.22	(0.89, 1.65)

Marital status				
Never been married	1.0		1.0	
Married/cohabiting	0.76^*∗*^	(0.59, 0.96)	0.82	(0.64, 1.05)
Divorced/separated or widowed	0.90	(0.70, 1.16)	1.04	(0.81, 1.34)
Others	0.46^*∗∗*^	(0.29, 0.74)	0.86	(0.53, 1.41)

HIV status				
Negative	1.0		1.0	
Positive	1.25^*∗∗*^	(1.09, 1.43)	1.25^*∗∗∗*^	(1.09, 1.43)
Unknown	0.72^*∗∗∗*^	(0.61, 0.85)	0.74^*∗∗*^	(0.62, 0.88)

^*∗∗∗*^ *p* < 0.001, ^*∗∗*^*p* < 0.01, and ^*∗*^*p* < 0.05.

**Table 4:** Proportion of OVC in a household with multiple OVCLHIV who all had their HIV status disclosed to them by their caregivers with different characteristics.

Caregiver characteristics	Proportion (%)	*p* value
Age (in years)		0.148
18–30	90.12	
31–60	94.40	
Above 60	95.45	

Sex		<0.001
Male	91.11	
Female	95.76	

Education level		0.006
Never attended	92.49	
Primary incomplete	98.13	
Primary complete	94.22	
Postprimary	98.32	

Marital status		0.012
Never been married	97.78	
Married/cohabiting	93.23	
Divorced/separated or widowed	95.49	
Others	90.00	

HIV status		0.820
Negative	94.01	
Positive	94.67	
Unknown	94.48	

**Table 5:** Characteristics associated with disclosure status of OVC living with caregivers of multiple OVCLHIV.

	Univariable (*n* = 2489)		Multivariable (*n* = 2489)	
	OR	95% CI	OR	95% CI
OVC characteristics				
Age (in years)				
8–10	1.0		1.0	
11–14	1.19	(0.83, 1.72)	1.21	(0.83, 1.76)
15 or above	1.12	(0.77, 1.62)	1.02	(0.69, 1.49)

Sex				
Male	1.0			
Female	1.20	(0.90, 1.60)		

School enrollment status				
Out of school	1.0			
In school	1.10	(0.76, 1.60)		

Location				
Rural	1.0		1.0	
Urban	2.01^*∗∗∗*^	(1.49, 2.70)	1.47^*∗*^	(1.05, 2.05)

Caregiver characteristics				
Age (in years)				
18–30	1.0			
31–60	1.18	(0.56, 2.49)		
Above 60	1.63	(0.72, 3.68)		

Sex				
Male	1.0		1.0	
Female	2.27^*∗∗∗*^	(1.69, 3.04)	1.97^*∗∗∗*^	(1.05, 2.05)

Education level				
Never attended	1.0		1.0	
Primary incomplete	2.66^*∗∗*^	(1.32, 5.36)	2.72^*∗∗*^	(1.32, 5.62)
Primary complete	1.42^*∗*^	(1.01, 1.99)	1.33	(0.92, 1.93)
Postprimary	7.42^*∗∗*^	(1.78, 30.98)	6.79^*∗∗*^	(1.58, 29.25)

Marital status				
Never been married	1.0		1.0	
Married/cohabiting	0.19^*∗∗*^	(0.07, 0.54)	0.30^*∗*^	(0.11, 0.84)
Divorced/separated or widowed	0.33^*∗*^	(0.12, 0.90)	0.37^*∗*^	(0.13, 1.03)
Others	0.20^*∗*^	(0.05, 0.86)	0.34	(0.08, 1.39)

HIV status				
Negative	1.0			
Positive	0.98	(0.69, 1.37)		
Unknown	1.03	(0.64, 1.65)		

^*∗∗∗*^ *p* < 0.001, ^*∗∗*^*p* < 0.01, and ^*∗*^*p* < 0.05.

## Data Availability

This study is based on data from the USAID Kizazi Kipya Project (2016–2021) in Tanzania. Pact Tanzania is the primary organization implementing the project and hence owns the data. The datasets analyzed during the current study are not publicly available due to confidentiality restrictions but are available from Pact Tanzania on reasonable request.

## Abbreviations

ART: Antiretroviral treatment
CHW: Community health worker
CI: Confidence interval
CTC: Care and treatment clinic
FCAA: Family and child asset assessment
GEE: Generalized estimating equations
HIV: Human immunodeficiency virus
OR: Odds ratios
OVC: Orphans and vulnerable children
OVCLHIV: Orphans and vulnerable children living with HIV
PLHIV: People living with HIV
UNAIDS: The Joint United Nations Programme on HIV/AIDS
USAID: United States Agency for International Development.
